# Supplementary material for: Causal interplay between lactose intolerance and gut microbiota: a combined bidirectional Mendelian randomization and in vivo validation study
Source: Front Nutr. 2026 Jun 1;13:1803337. doi: 10.3389/fnut.2026.1803337 (PMC13265576; doi:10.3389/fnut.2026.1803337)
Supplement: Supplementary file 1 [file Data_Sheet_1.zip › supplementary materials/Forward/leave one out cross validation/ebi-a-GCST90027629.finngen_R12_E4_LACTONAS.pdf]

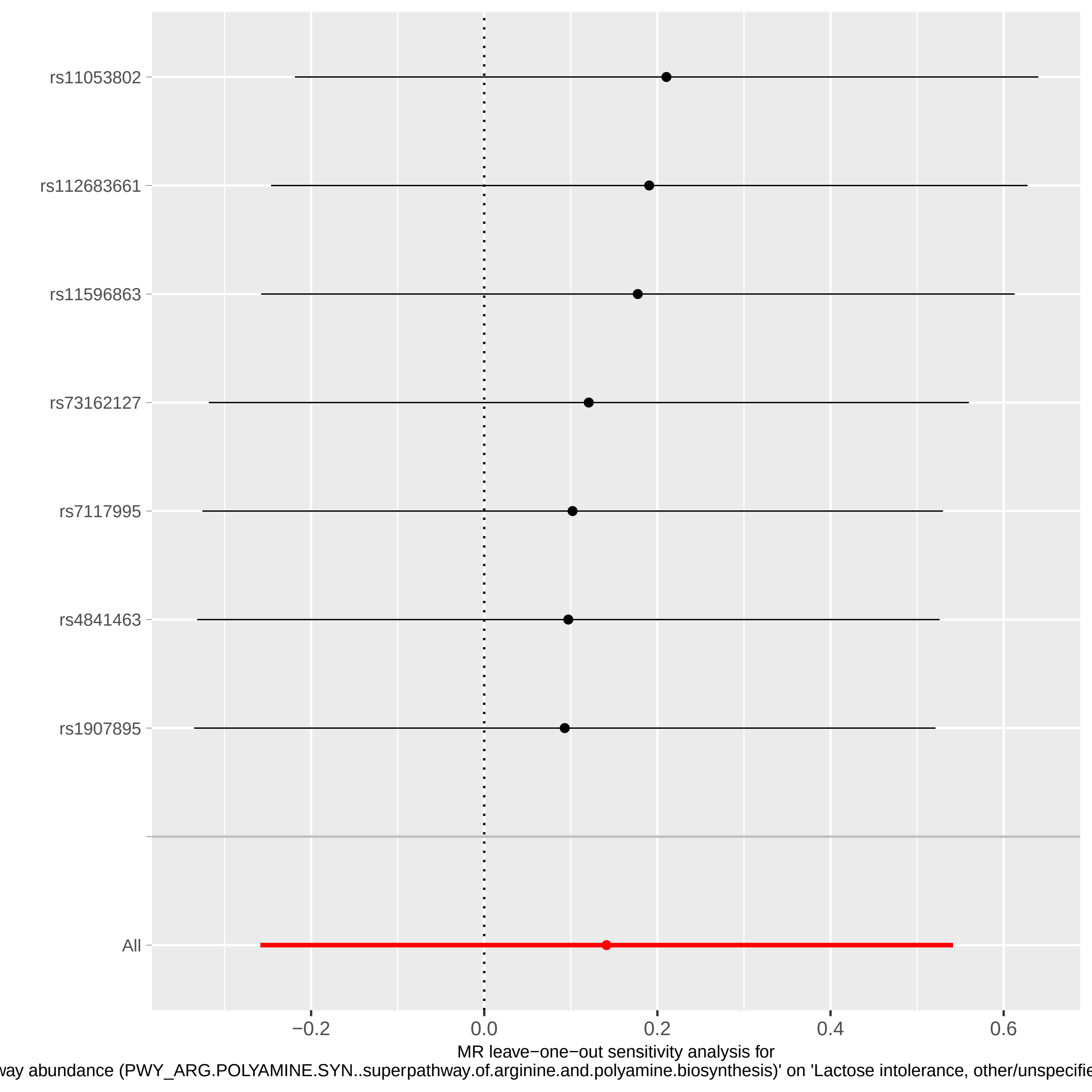
rs11053802
rs112683661
rs11596863
rs73162127
rs7117995
rs4841463
rs1907895
All
−0.2
0.0
0.2
0.4
0.6
MR leave−one−out sensitivity analysis for
way abundance (PWY_ARG.POLYAMINE.SYN..superpathway.of.arginine.and.polyamine.biosynthesis)' on 'Lactose intolerance, other/unspecifie
